# Effects of Supplementing the Diets of Mice With Different Concentrations of Conjugated Linoleic Acid on the Anti‐Inflammatory Function of the Mammary Gland

**DOI:** 10.1002/fsn3.72367

**Published:** 2026-10-01

**Authors:** Zheming Ren, Wenqi Liang, Hang Zhang, Minghui Liu, Yuqiong Wang

**Affiliations:** ^1^ College of Animal Science and Technology Inner Mongolia Minzu University Tong Liao People's Republic of China

**Keywords:** anti‐inflammatory, antioxidant, CLA, mammary gland, mice

## Abstract

In this study, we evaluated the impact of the dietary addition of different concentrations of conjugated linoleic acid (CLA) on the antioxidant capacity and anti‐inflammatory effects of serum and breast tissue in mice. Twenty‐four ICR mice (BW = 37.79 ± 2.17 g) at 7 days of pregnancy were divided into four groups (*n* = 6 per group): a control (CN) group, which was fed a normal diet, and three groups fed a normal diet supplemented with 0.5% CLA, 1% CLA, or 2% CLA. Each mouse had free access to food and water. After 49 days of feeding, mice were injected with lipopolysaccharide (LPS) to induce inflammation, and 24 h later, the experimental animals were anesthetized by intraperitoneal injection of pentobarbital sodium (40–50 mg/kg). After achieving deep anesthesia, blood was collected from the posterior orbital venous plexus. Subsequently, an excessive dose of pentobarbital sodium (total dose of 150–200 mg/kg) was administered to euthanize the animals. After confirming the cessation of breathing and heartbeat, breast tissues were collected for analysis. The expression levels of antioxidant and inflammatory factors in serum and mammary gland tissues were measured by ELISA, and the mRNA expression levels of mammary inflammatory factors were further quantified by RT‐PCR. Parts of the tissue samples were retained for histopathological observation. The results showed that compared with the control treatment, CLA supplementation significantly increased both the total antioxidant capacity (T‐AOC) and the levels of superoxide dismutase (SOD), with the 1% CLA group exhibiting the most pronounced increase (*p* < 0.001). The contents of interleukin 1β (IL‐1β), tumor necrosis factor (TNF‐α) and interleukin 6 (IL‐6), decreased significantly, with the lowest levels in the 1% CLA group (*p* < 0.001). Both MDA and GPx levels showed significant decline, with the lowest levels in the 1% CLA group (*p* < 0.001). The relative expression of the PPAR‐γ gene (*p* < 0.05) was significantly higher in the 2% CLA group than in the CN group, and the 1% CLA group showed certain promoting effect. Compared with the CN group, the relative expression levels of the TNF‐α and IL‐8 genes were significantly decreased in the 1% CLA group (*p* < 0.05). Histopathological analysis revealed that the mammary acinar structure of mice in the 1% CLA group remained intact, with minimal inflammatory infiltration in the acinar lumen. In conclusion, the dietary addition of CLA exerted marked antioxidant and anti‐inflammatory effects in the serum and mammary gland tissues of mice, with the best results observed with the 1% supplementation level. This study focuses on the dose optimization of CLA, and the results provide a foundational dose reference for the translational application of CLA in dairy cow farming.

## Graphical abstract

Dietary conjugated linoleic acid (CLA), a functional fatty acid, exhibits antioxidant and anti‐inflammatory activities. This study assessed its protective effects against mammary gland inflammation in LPS‐stimulated mice. 1% CLA supplementation yielded the optimal protective effect, offering a reference for the prevention of dairy cow mastitis.

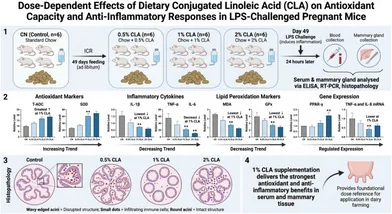

**Abbreviations**
CLA: conjugated linoleic acid
GPx: glutathione peroxidase
LPS: lipopolysaccharide
MDA: malondialdehyde
SOD: superoxide dismutase
T‐AOC: total antioxidant capacity

## 1 Introduction

The continuous development of intensive livestock farming has significantly improved the production performance of dairy cows in China. However, oxidative stress resulting from enhanced mammary metabolism has become increasingly prominent (Sordillo et al. 2007; Sordillo and Aitken 2009). Excessive free radical production can lead to poor feed utilization and reduced milk yield in cows (Stefanon et al. 2002), rendering them more susceptible to oxidative stress‐induced damage. This, in turn, leads to the occurrence of mastitis and related pathologies, which severely diminish milk quality and yield, resulting in substantial economic losses. This highlights the importance of the prevention and treatment of mastitis for maintaining dairy cow health and promoting the development of the dairy industry. Antibiotics have historically been the primary intervention for the prevention and treatment of mastitis in dairy cows; however, issues involving antibiotic resistance and residues have become increasingly severe. Because prevention remains the most effective control method, it is urgent to identify dietary additives that reduce oxidative stress and improve anti‐inflammatory capacity in mammary tissue to enhance overall production efficiency.

Conjugated linoleic acid (CLA) comprises a group of positional and geometric isomers of linoleic acid characterized by a conjugated double bond structure. Current literature indicates that CLA is primarily found in meat (animal fats) and dairy products. Its content in vegetable oils is either considerably lower than in these sources or requires further processing to obtain. The dominant isomer is cis‐9, trans‐11‐CLA (c9, t11‐CLA), which comprises 80% to 90% of the total CLA content in milk (Chin et al. 1992). CLA, as a long‐chain unsaturated fatty acid, participates in a series of physiological activities within the body and possesses numerous important physiological functions such as anti‐cancer, anti‐inflammatory (Haghighatdoost et al. 2018), anti‐tumor (Kim et al. 2015), body fat reduction, and blood sugar reduction (Kim et al. 2016; Reza Rahbar et al. 2017). Research suggests that the dietary supplementation of c9, t11‐CLA in murine models of Alzheimer's disease effectively increases the expression of anti‐inflammatory cytokines such as interleukin‐10 (IL‐10) and IL‐19, thereby mitigating neuroinflammation (Fujita et al. 2021). Dietary supplementation with CLA significantly upregulates the mRNA expression of IL‐10 and promotes the production of anti‐inflammatory cytokines (Kong et al. 2018). One study found that CLA attenuates mitochondrial autophagy and inflammatory responses in bovine hepatocytes via the DRP1/mtDNA/STING pathway, thereby alleviating oxidative damage (Xie et al. 2024). Furthermore, studies have found that the dietary inclusion of rumen‐protected CLA alleviates mild systemic inflammation and oxidative stress in postpartum cows (Bayat et al. 2022). During the late lactation period, CLA supplementation increases milk protein content and improves maternal energy status. At the same time, the increase in citrate concentration in the CLA milk indicates a reduction in fatty acid synthesis in the mammary glands (Vogel et al. 2020). CLA also serves as a potent activator of antioxidant signaling pathways, promoting the production of endogenous antioxidant enzymes and thereby exerting antioxidant effects. Studies have found that high milk yield during early lactation is closely associated with oxidative stress, and feeding a certain dose of CLA in early lactation can improve the activity of antioxidant enzymes in serum (Haubold et al. 2020). However, in the middle lactation period of cows, CLA had a significant effect on energy balance and milk composition, but the antioxidant effect was not significant (Basiricò et al. 2017). Studies have shown that CLA can regulate the production of inflammatory response‐related cytokines, mainly including interleukin 6 (IL‐6), tumor necrosis factor α (TNF‐α) and interleukin 1 β (IL‐1β) (Cuciniello et al. 2024). Notably, dietary CLA can reduce the levels of oxidative stress markers and pro‐inflammatory cytokines (TNF‐α and IL‐6) in the cerebral cortex of mice (Mohammadi et al. 2020). Furthermore, in vitro, the addition of c9, t11‐CLA and t10, c12‐CLA isomers inhibited both the apoptosis of peripheral blood mononuclear cells and the production and expression of TNF‐α, IL‐1β, and IL‐6 (Valenzuela et al. 2023).

Our previous study used bovine mammary epithelial cells (BMECs) as a model to compare the anti‐inflammatory effects of different concentrations of c9, t11‐CLA, and we found that 200 μmol/L c9, t11‐CLA effectively inhibited the production of pro‐inflammatory factors in BMECs, providing a cellular experimental basis for the subsequent in vivo application of CLA. This study takes LPS‐stimulated pregnant mice as the in vivo animal model, with the core goal of optimizing the dietary supplementation dose of CLA to systematically evaluate the antioxidant and anti‐inflammatory effects of different concentrations of CLA on serum and mammary gland tissues of mice, and screen out the optimal effective dose of CLA for alleviating mammary gland inflammation. This study focuses on dose optimization and in vivo efficacy verification; the results are expected to provide direct in vivo experimental data and a practical dose reference for the subsequent application of CLA as a dietary additive in dairy cow breeding.

## 2 Materials and Methods

### 2.1 Animals and Treatments

Twenty‐four C57BL/6J ICR female mice, weighing 37.79 ± 2.17 g, were obtained from the Academy of Military Medical Sciences (Beijing, China) [approval number: SCXK (Jing) 2019‐0010]. CLA was provided by Aohai Biological Co. Ltd. (Qingdao, China). The purity of the CLA was 80.4% (38.5% cis9, trans11‐CLA, 38.3% trans10, cis12‐CLA, and 3.6% other CLA isomers, with the remainder of the preparation consisting of 10.8% oleic acid, 2.8% stearic acid, 3.1% palmitic acid, and 1.5% linoleic acid; 1.4% was unknown). The feed for mice was purchased from SPF Biotechnology Co. Ltd. (Beijing, China). The study protocol was reviewed and approved by the Animal Care and Ethics Committee of Inner Mongolia Minzu University, Tongliao, China (NO. NMD‐DW‐2024‐11‐15). During the experiment, the animals were housed in cages under controlled laboratory conditions (air temperature of 22°C ± 2°C, 60%–70% relative humidity, and a 12‐h/12‐h light/dark cycle) with ad libitum access to feed and water. ICR mice that have been pregnant for 7 days, the mice were randomly divided into four groups (*n* = 6/group): a normal diet (CN) group, a normal diet + 0.5% CLA (0.5% CLA) group, a normal diet + 1% CLA (1% CLA) group, and a normal diet + 2% CLA (2% CLA) group. Each pregnant mouse was housed in a single cage. The different proportions of CLA were pre‐added to the normal diet to product pellet feed. There was no significant difference in the feed intake among the different groups of mice. The details are shown in Table 1. After 7 weeks, 0.2 mg/mL LPS (L2630, Sigma, USA) 50 μL/mice was injected into these mice via the fourth mammary pair after anesthesia, 24 h after the injection of LPS. The process of euthanizing the mice followed the previously described method of Laferriere et al. (Laferriere et al. 2020), with minor modifications. The experimental animals were anesthetized by intraperitoneal injection of pentobarbital sodium (40–50 mg/kg). After achieving deep anesthesia, blood was collected from the posterior orbital venous plexus. The collected blood sample was used for subsequent analysis of detection indicators. Then, an excessive dose of pentobarbital sodium (total dose of 150–200 mg/kg) was administered to euthanize the animals, and the cessation of breathing and heartbeat was confirmed afterward. The mammary gland tissue were divided into two parts: one part was fixed in a 10% formalin solution, while the other part was stored at −80°C for subsequent experiments.

**TABLE 1:** The average food intake of mice.

Group	CN group	0.5% CLA group	1% CLA group	2% CLA group	*p*
Average feed intake(g)	14.67 ± 1.12	14.37 ± 1.23	14.79 ± 1.24	14.52 ± 1.14	0.982

### 2.2 Measurement of Inflammatory Factor Levels and Antioxidant Enzyme Activities by ELISA

Following euthanasia, blood was taken from the eyes and stored at 4°C for 4 h. The blood was subsequently centrifuged, and the supernatant, comprising serum, was frozen at −20°C for subsequent analysis. The serum or mammary tissue frozen in liquid nitrogen was mixed with 9 times the volume of pre‐cooled deionized water at a ratio of weight (g):volume (mL) = 1:9. After homogenization in an ice‐cold water bath, the samples were centrifuged at 4000 rpm for 10 min, and the supernatant was collected. The concentrations of the pro‐inflammatory cytokines TNF‐α, IL‐6, and IL‐1β in the supernatant were determined using the respective ELISA kits (Shanghai Enzyme‐linked Biotechnology Co. Ltd), and also measured the levels of malondialdehyde (MDA), glutathione peroxidase (GPx), SOD and T‐AOC in the serum.

### 2.3 Real‐Time Fluorescence PCR

Mammary tissue was retrieved from −80°C storage, ground in liquid nitrogen, and lysed in 1 mL of RNAiso for RNA extraction. The resulting RNA was dissolved in enzyme‐free water, and its concentration and purity were determined using an ultramicro‐spectrophotometer. RNA integrity was confirmed by agarose gel electrophoresis. The extracted RNA was then reverse‐transcribed into cDNA using a cDNA kit. According to the reagent TB Green Premix Ex TaqTMII, a total of 20 μL was prepared by sequentially adding the reagents to prepare the PCR reaction solution. The reaction program for performing real‐time PCR was as follows: a 30s pre‐denaturation at 95.0°C, a 30s denaturation at 95.0°C, and a 20s of annealing at the *T*_m_ value, with a total of 40 cycles. GAPDH was used as an internal reference gene. The specific primer sequences can be found in Table 2. The primers were synthesized by Sangon Biotech (Shanghai) Co. Ltd.

**TABLE 2:** Primer sequence of the tested gene.

Gene	Primer sequences
TNF‐α	F:ACGGCATGGATCTCAAAGAC R:GTGGGTGAGGAGCACGTAGT
GAPDH	F:TCAACGGGAAGCTCACTGG R:CCCCAGCATCGAAGGTAGA
IL‐1β	F:TGTGATGTTCCCATTAGAC R:AATACCACTTGTTGGCTTA
PPARγ	F:CACAATGCCATCAGGTTTGG R:GCTGGTCGATATCACTGGAGATC

### 2.4 Preparation of Mammary Gland Pathological Sections and HE Staining

Mice were euthanized by intraperitoneal injection of excessive pentobarbital sodium, we removed the mammary glands from their abdominal walls using surgical scissors and washed them with physiological saline. After fixation in a 10% formalin solution for 24 h, the tissues were placed in a dehydration box, rinsed with running water, and dehydrated through a graded ethanol series. Next, the tissues were cleared in xylene I and xylene II, paraffin‐embedded, sectioned, dried, and dewaxed. Following standard HE staining, the sections were mounted and imaged under an electron microscope.

### 2.5 Statistical Analysis

We sorted out the data using Excel software. We conducted an in‐depth analysis of the collected experimental data by using one‐way ANOVA LSD function in IBM SPSS 27 software. Among multiple samples in the multiple comparisons, a one‐way analysis of variance (ANOVA) was employed and comparisons were made between the groups. The results showed that *p*‐value less than 0.001 indicated a highly significant difference, *p*‐value less than 0.05 indicated a significant difference, and *p*‐value greater than 0.05 indicates that the difference was not significant. We employed the $2^{-∆∆C_{t}}$ method to estimate the relative expression level of genes.

## 3 Results

### 3.1 The Effects of CLA on Antioxidant Enzyme Activities and Inflammatory Cytokine Levels in Mouse Serum

As shown in Table 3, compared with the CN, the contents of MDA and GPx significantly decreased in the all CLA groups, which 0.5% CLA group was the highest, followed by 2% CLA group, and 1% CLA group were the lowest (*p* < 0.001), decreasing by about 3.91% and 5.49% respectively. Compared with the CN, SOD and T‐AOC in each CLA group were significantly increased, which the 0.5% CLA group were the lowest, followed by 2% CLA group, and 1% CLA group was the highest (*p* < 0.001), which were higher 7.38% and 7.13%, respectively. The results indicated that the antioxidant capacity of serum in mice was significantly improved by adding 1% CLA to the diet.

**TABLE 3:** Effects of different CLA concentrations on antioxidant enzymes in blood.

Group	CN group	0.5% CLA group	1% CLA group	2% CLA group	*p*
MDA (nmol/mL)	6.75 ± 0.08^a^	5.52 ± 0.23^b^	4.61 ± 0.24^c^	2.84 ± 0.29^d^	< 0.001
SOD (U/mg)	6.27 ± 0.38^d^	8.35 ± 0.41^c^	10.34 ± 0.54^b^	13.65 ± 0.21^a^	< 0.001
T‐AOC (mmol/L)	7.94 ± 0.59^d^	10.19 ± 0.46^c^	12.28 ± 0.63^b^	15.07 ± 0.51^a^	< 0.001
GPx (U/L)	9.09 ± 0.37^a^	7.38 ± 0.38^b^	3.60 ± 0.28^d^	5.46 ± 0.05^c^	< 0.001

*Note:* Values within the same row sharing no common lowercase superscript letters differ significantly (*p* < 0.05) or extremely significantly (*p* < 0.01). Values with identical or no superscript letters show no significant difference (*p* > 0.05).

Compared with the CN group, the levels of IL‐1β, TNF‐α, and IL‐6 were significantly reduced in all CLA treatment groups, with the 1% CLA group showing the lowest values (decreases of 56.28%, 69.68%, and 40.93%, respectively; *p* < 0.001). These findings suggested that the 1% CLA concentration exerted the most significant anti‐inflammatory effect in the serum of mice (Table 4).

**TABLE 4:** The influence of different CLA concentrations on serum inflammatory factors.

Group	CN group	0.5% CLA group	1% CLA group	2% CLA group	*p*
IL‐1β (pg/mL)	89.81 ± 0.65^a^	78.96 ± 3.25^b^	33.53 ± 3.77^d^	57.18 ± 1.37^c^	< 0.001
TNF‐α (pg/mL)	130.69 ± 5.47^a^	113.98 ± 5.60^b^	61.01 ± 5.35^d^	94.56 ± 5.53^c^	< 0.001
IL‐6 (pg/mL)	68.41 ± 2.70^a^	59.03 ± 1.01^b^	27.48 ± 2.33^d^	45.19 ± 1.64^c^	< 0.001

*Note:* Values within the same row sharing no common lowercase superscript letters differ significantly (*p* < 0.05) or extremely significantly (*p* < 0.01). Values with identical or no superscript letters show no significant difference (*p* > 0.05).

### 3.2 The Effects of CLA on the Activities of Antioxidant Enzymes and the Levels of Inflammatory Cytokines in Mouse Mammary Tissues

According to Table 5, compared with the CN group, the levels of MDA and GPx in each experimental group significantly decreased. Among them, the 2% CLA group had the highest level, the 0.5% CLA group had the second highest, and the 1% CLA group had the lowest (*p* < 0.001), decreasing by about 3.56% and 4.09%, respectively. Compared with the CN group, the SOD and T‐AOC levels in each experimental group were increased significantly. Among them, the 0.5% CLA group had the lowest level, the 2% CLA group had the second lowest, and the 1% CLA group had the highest (*p* < 0.001), with increases of approximately 5.71% and 6.29%, respectively.

**TABLE 5:** Effects of different CLA concentrations on antioxidant enzymes in mice mammary tissue.

Group	CN group	0.5% CLA group	1% CLA group	2% CLA group	*p*
MDA (nmol/mL)	4.85 ± 0.16^a^	3.14 ± 0.04^c^	1.29 ± 0.16^d^	3.98 ± 0.05^b^	< 0.001
SOD (U/mg)	3.01 ± 0.27^d^	4.33 ± 0.21^c^	8.72 ± 0.16^a^	5.56 ± 0.26^b^	< 0.001
T‐AOC (mmol/L)	3.76 ± 0.26^d^	5.35 ± 0.27^c^	10.05 ± 0.16^a^	6.71 ± 0.17^b^	< 0.001
GPx (U/L)	6.27 ± 0.26^a^	4.32 ± 0.20^c^	2.18 ± 0.07^d^	5.19 ± 0.07^b^	< 0.001

*Note:* Values within the same row sharing no common lowercase superscript letters differ significantly (*p* < 0.05) or extremely significantly (*p* < 0.01). Values with identical or no superscript letters show no significant difference (*p* > 0.05).

We can observe that in each experimental group, the contents of IL‐1β, TNF‐α, and IL‐6 have decreased significantly. The lowest contents of these cytokines were found in the 1% CLA group, showing decreases of approximately 16%, 34%, and 13%, respectively (*p* < 0.001), followed by the 2% CLA group (*p* < 0.001). Our results implied that the 1% CLA inclusion level exerted the most significant anti‐inflammatory effect on the mouse mammary gland (Table 6).

**TABLE 6:** Effect of different CLA concentrations on inflammatory factors in the breast.

Group	CN group	0.5% CLA group	1% CLA group	2% CLA group	*p*
IL‐1β (pg/mL)	62.05 ± 0.62^a^	42.51 ± 2.51^c^	16.00 ± 1.87^d^	54.36 ± 2.15^b^	< 0.001
TNF‐α (pg/mL)	95.04 ± 2.83^a^	68.25 ± 1.92^c^	34.86 ± 3.50^d^	82.73 ± 0.83^b^	< 0.001
IL‐6 (pg/mL)	48.37 ± 0.40^a^	32.67 ± 1.77^c^	13.96 ± 1.51^d^	39.50 ± 1.13^b^	< 0.001

*Note:* Values within the same row sharing no common lowercase superscript letters differ significantly (*p* < 0.05) or extremely significantly (*p* < 0.01). Values with identical or no superscript letters show no significant difference (*p* > 0.05).

### 3.3 The Influence of CLA on the Expression Levels of Inflammatory Factors in Mice Mammary Tissues

As displayed in Figure 1, compared with the CN group, the relative expression levels of the PPARγ gene were significantly increased in the 2% CLA group (*p* < 0.05), while those in the 1% CLA group exhibited an increasing trend. Additionally, the relative expression level of the TNF‐α gene was significantly lower in both the 0.5% CLA group and 1% the CLA group than in the CN group (*p* < 0.05), with the 1% CLA group displaying the largest decrease. Meanwhile, the relative expression level of IL‐8 was significantly downregulated in both the 1% CLA and 2% CLA groups relative to that in the CN group (*p* < 0.05). Again, the most pronounced change was observed in the 1% CLA group. The results showed that the anti‐inflammatory effect on the mammary gland of mice was most significant when a 1% CLA concentration was added to the feed.

**FIGURE 1:**
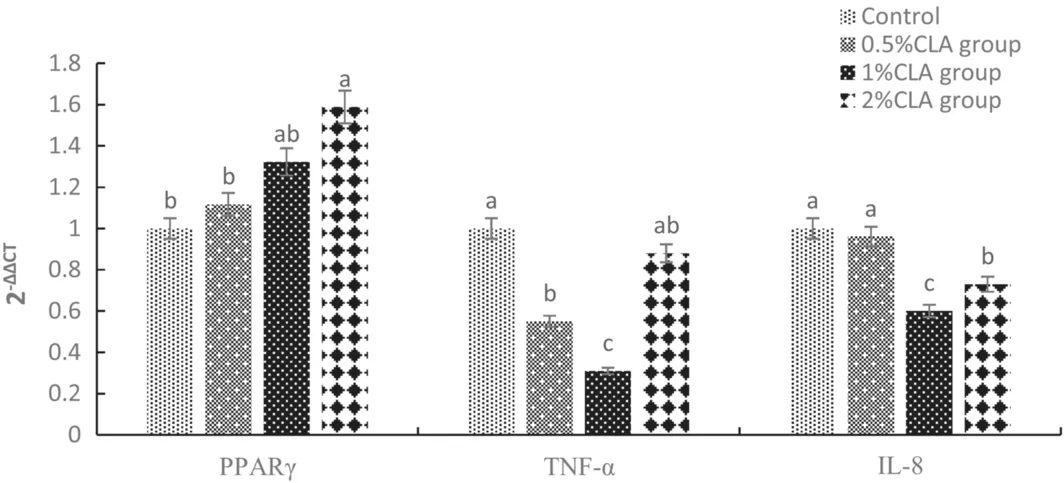
Effect of CLA on the relative expression levels of PPAR‐γ, TNF‐α and IL‐8 in mammary gland of mice. Values with different superscript letters within the same gene indicate significant differences (*p* < 0.05).

### 3.4 Histopathological Evaluation of CLA on LPS‐Induced Mammary Gland Inflammatory Damage in Mice

As illustrated in Figure 2A, while the acinar structure of mammary tissue in the CN group remained intact, the mammary tissue displayed marked swelling, accompanied by inflammatory cell infiltration. In the 0.5% CLA group, the mammary acini also remained intact; however, most acini exhibited noticeable inflammatory infiltration (Figure 2B). In mice with 1% CLA, the structure of the mammary acini remained intact, with only a small amount of inflammatory infiltration within the acini, and most glands in the 1% CLA group were normal (Figure 2C). Similarly, the 2% CLA group retained intact acinar structures, though a small amount of inflammatory infiltration persisted in most glandular tissue (Figure 2D). The CN group showed typical mastitis lesions and successfully replicated the model. These findings suggested that all CLA dosages enhanced the anti‐inflammatory capacity of the mouse mammary gland to varying degrees, with the 1% CLA inclusion level providing the most pronounced protective effect.

**FIGURE 2:**
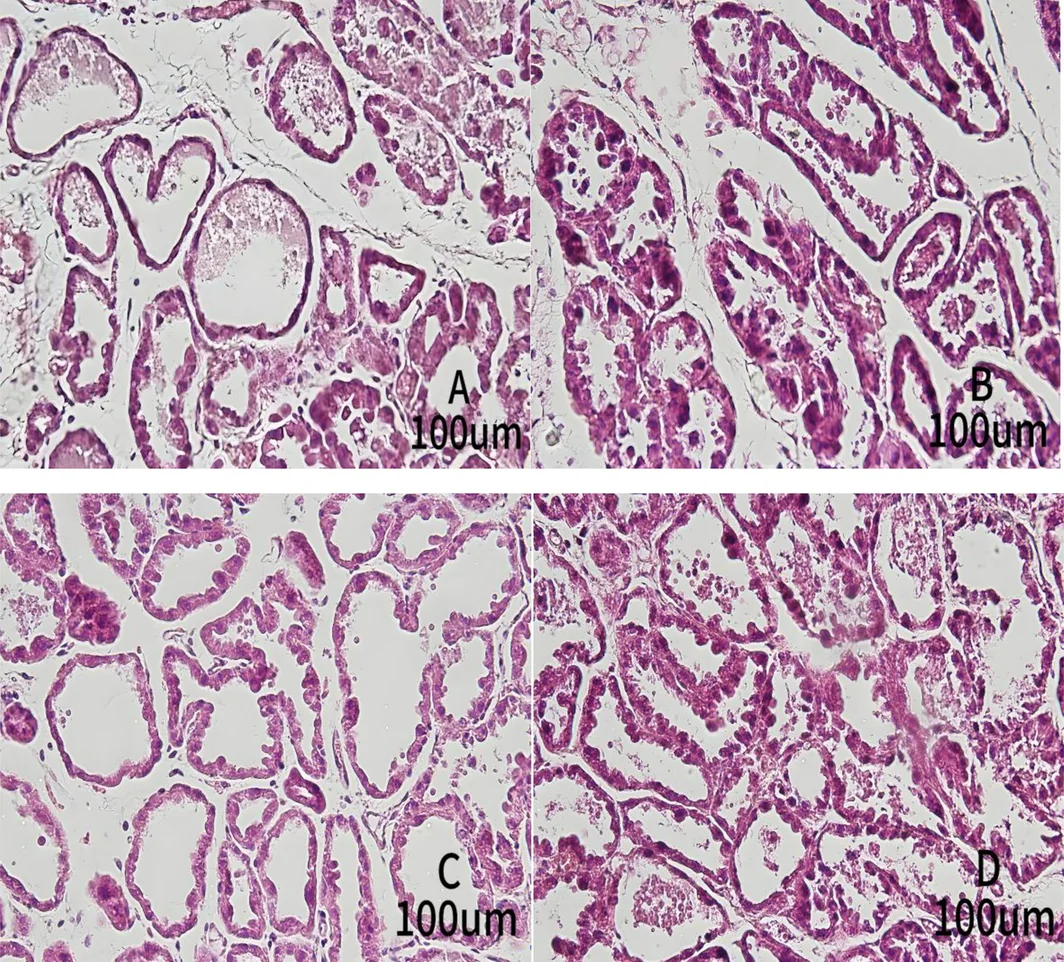
Histological observation of mammary gland tissue in mice. (A) Control group; (B) 0.5% CLA group; (C) 1% CLA group; (D) 2% CLA group. Scale bar = 100 μm.

## 4 Discussion

Oxidative stress occurs when the dynamic equilibrium between an organism's pro‐oxidant and antioxidant systems is disrupted. This imbalance leads to the excessive production of reactive oxygen species (ROS), which ultimately compromises cellular structure and function (Jebur et al. 2016). Oxidative stress can be quantified by measuring the levels of biomarkers such as GPx, MDA, and SOD. Numerous investigations have confirmed that CLA possesses potent antioxidant properties. However, evidence indicates that CLA exhibits isomer‐specific differences in the modulation of GPx activity. For instance, Zhang et al. (2024) demonstrated that c9, t11‐CLA effectively modulates GPx activity in BMECs, thus enhancing overall antioxidant capacity. Consistent with the findings of this study, the GPx activity in the CLA supplementation groups was significantly lower than that in the control group. The research found that a study conducted on patients with atherosclerosis showed that after daily supplementation with 3 g of a 50:50 ratio of c9, t11 and t10, c12 CLA isomers forms for 2 months, the GPx levels significantly increased (Eftekhari et al. 2013), which is contrary to the results of this study. It has been reported that CLA reduces MDA levels in humans without impacting GPx activity (Suksatan et al. 2022). This indicates that the effect of adding CLA is variable, ranging from significant to insignificant. There is still some controversy regarding this. These results suggest that decreased GPx activity may not indicate impaired antioxidant capacity. It may reflect the reduction in oxidative stress due to the inhibition of LPS‐induced oxidative stress mediated by CLA, thereby lowering the oxidative burden of the body. Evidence suggests that CLA improves the redox status in BMECs, particularly t10, c12‐CLA, which exhibits pronounced protective effects against oxidative stress (Basiricò et al. 2015). Furthermore, a 50:50 mixture of the c9, t11‐CLA and t10, c12‐CLA isomers, which influences the protein abundance of heme oxygenase‐1 (HMOX‐1), an antioxidant enzyme, was reported to enhance cellular resistance to oxidative stress in bovine peripheral blood mononuclear cells (PBMCs) (Ávila et al. 2024). Compared with essential fatty acids (EFAs), CLA is more effective in enhancing the antioxidant capacity of BMECs and alleviating oxidative damage (Basiricò et al. 2017). Lower concentrations of MDA, a primary biomarker of lipid peroxidation, correlate with increased antioxidant capacity; conversely, elevated MDA levels reflect excessive ROS generation (Karimi et al. 2024). Prior research has demonstrated that dietary CLA supplementation significantly reduces MDA content and lipid peroxidation (Basiricò et al. 2017; Ayala et al. 2014). Here, we found that the levels of both markers were significantly decreased in the CLA supplementation groups relative to those in the CN group. Specifically, our data indicated that the 1% CLA group exhibited the lowest levels of both markers, suggesting that this dietary inclusion dosage provides the most pronounced antioxidant effect in murine mammary tissue.

T‐AOC reflects the overall antioxidant defense status of an organism. The brown rat maintains T‐AOC stability by upregulating the activity of antioxidant enzymes such as SOD, which facilitates the elimination of excessive ROS and prevents lipid peroxidation (Xu et al. 2019). In this study, the significant increase in SOD activity observed in the CLA supplementation groups is consistent with previous findings. For instance, Aydın et al. (Aydın et al. 2018) found that CLA upregulates the levels of antioxidant enzymes, such as GSH, in mice, confirming that it plays a protective role against oxidative stress. Furthermore, one study reported that CLA‐rich butter significantly improves SOD activity in mice and reduces the extent of cell damage, suggesting that CLA‐derived lipids enhance the overall antioxidant capacity of mice (Chinnadurai et al. 2013). Chen et al. (2024) demonstrated that dietary supplementation with 1%–3% CLA in Hailan Brown laying hens in the late laying period markedly improves SOD activity, while 2%–4% CLA bolsters T‐AOC (Chen et al. 2024). Consistent with these reports, our data showed that both SOD activity and T‐AOC were significantly increased in the CLA treatment groups relative to those in the CN group, suggesting that CLA treatment improved the antioxidant capacity of mice. Notably, the highest values for both indicators were recorded with the 1% CLA dietary supplementation. The results of pro‐inflammatory cytokines and inflammation‐related gene expression in this study also did not show a monotonic trend with increasing CLA concentration: the 0.5% CLA only slightly downregulated IL‐1β, TNF‐α, and IL‐8; the inhibitory effect of the 2% CLA on pro‐inflammatory factors was significantly weaker than that of the 1% group, with only the 1% CLA achieving maximum suppression of TNF‐α and IL‐8 transcription and protein secretion. The mRNA expression level of the PPARγ gene was highest in the 2% CLA group, yet the anti‐inflammatory effects did not increase correspondingly. Since this study only measured PPARγ gene expression without pathway‐level validation, it is speculated that dose‐dependent saturation or negative feedback regulation within the PPARγ signaling pathway may occur. At a 0.5% CLA, activation of PPARγ was insufficient to effectively block NF‐κB nuclear translocation and subsequent inflammatory cascade reactions. In contrast, 1% CLA achieved moderate PPARγ activation, thereby maximizing the inhibition of downstream pro‐inflammatory factor release without triggering negative feedback. Histopathological examination of the mammary tissue further supported this dose–response pattern: significant inflammatory cell infiltration remained in the acini of both the 0.5% and 2% CLA groups, whereas the 1% CLA group exhibited intact acinar structures with minimal infiltration. This further confirms that CLA alleviates LPS‐induced mammary inflammation in a dose‐specific manner, without a linear dose–response relationship.

During the gestational and peak lactation phases, the mammary glands are highly susceptible to oxidative stress, which may predispose cows to mastitis. This condition reduces both milk production and quality, potentially causing irreversible tissue damage if not promptly addressed. In our study, we noted that following LPS stimulation, mice in the CN group displayed incomplete mammary acini and significant inflammatory infiltration. Conversely, histopathological analysis suggested that the structure of the mammary acini in the 1% CLA group remained intact, with only minimal inflammatory infiltration detected. This protective outcome provides a reference for further exploration of the modulatory effect of CLA on mammary inflammation. The induction of inflammation exacerbates oxidative stress, functioning as a primary catalyst for its progression. IL‐1β, a central pro‐inflammatory cytokine, serves as the key medium of the damage. The release of IL‐1β stimulates the production of reactive oxygen species (ROS) in epithelial cells, while concomitantly reducing the activity of antioxidant enzymes such as GPx and SOD, thus exacerbating tissue damage (Ferrero‐Miliani et al. 2007). Extensive research has indicated that CLA possesses significant potential in mitigating inflammation. Specifically, markers such as IL‐1β, TNF‐α, and IL‐6 as a marker of important pro‐inflammatory cytokines (Yang et al. 2025). In a human trial, it was found that CLA increases serum levels of the anti‐inflammatory cytokine IL‐10 while concomitantly reducing those of NF‐κB, TNF‐α, IL‐2, and IL‐8 (Penedo et al. 2013). Our data show that the relative expression levels of the Tnfa and Il8 genes were significantly decreased in the 1% CLA group compared with those of the CN group. Notably, Tnfa expression reached its lowest level in this group, consistent with previous findings. Bassaganya‐Riera et al. (2012) suggested that CLA inhibits the production of TNF‐α and IL‐17 in peripheral CD4+ and CD8+ T‐cell subpopulations (Bassaganya‐Riera et al. 2012). In BMEC models, c9, t11‐CLA has been shown to counteract *Escherichia coli* ‐induced inflammation by suppressing the overexpression of pro‐inflammatory factors such as IL‐1, IL‐6, and IL‐8, while upregulating the production of anti‐inflammatory cytokines (Ma et al. 2019). Additionally, treatment with 50 μM c9, t11‐CLA and t10, c12‐CLA was shown to inhibit pro‐inflammatory cytokine transcription in BMECs (Dipasquale et al. 2018), thereby limiting the release of pro‐inflammatory factors and subsequent ROS production (Ma et al. 2021).

The primary function of PPARγ primarily involves metabolic‐inflammatory regulation (Mandard and Patsouris 2013), PPARγ interacts with NF‐κB, preventing its nuclear translocation and suppressing the production of TNF‐α, IL‐1, and IL‐6 (Yu et al. 2002). Moreover, Yu et al. (Shuai et al. 2025) found that CLA downregulates the mRNA levels of IL‐1β, TNF‐α, and IL‐6 in RAW264.7 macrophages via the PPARγ pathway. In the current work, cytokine levels were significantly lower in the CLA supplementation groups than in the CN group, thus achieving anti‐inflammatory effects. Studies have further shown that the activation of PPARγ is the core mechanism by which CLA alleviates colitis (Bassaganya‐Riera et al. 2004; Hontecillas et al. 2003; Guangxian et al. 2018). In atherosclerosis models, CLA‐mediated PPARγ activation inhibits pro‐inflammatory cytokine expression and promotes the polarization of macrophages toward an anti‐inflammatory phenotype (Dipasquale et al. 2018). Manna and Ramesh (2005) found that 50 μmol/L c9, t11‐CLA significantly reduces the mRNA levels of IL‐6, TNF‐α, and the anti‐inflammatory factor IL‐10 in LPS‐stimulated BMECs. While TNF‐α typically drives cytokine production by activating NF‐κB (Edwards et al. 2009; Wong and Tergaonkar 2009; Su et al. 1999), evidence supports that PPARγ can inhibit NF‐κB through an IκB‐α‐depedent mechanism, which effectively reduces the levels of pro‐inflammatory cytokines. However, it should be emphasized that our study only detected the mRNA level of PPARγ, and did not verify the activation state of PPARγ protein, the binding of PPARγ to NF‐κB, or the downstream signaling changes. Therefore, this study only provides a preliminary expression trend suggesting the potential involvement of PPARγ in CLA's mammary anti‐inflammatory effect, and cannot confirm the direct causal relationship and specific regulatory mechanism between PPARγ and CLA's anti‐inflammatory action. In conclusion, these results suggest that among the three dosages tested, dietary CLA at a concentration of 1% yields the most significant anti‐inflammatory effect in the mammary glands of mice.

To assess the potential practical value of the study's findings, we compared the CLA intake in mice with actual dairy cow production. In this study, the 1% dietary CLA concentration in mice. For C57BL/6J male mice with an average body weight of 37.79 g and a standard daily feed intake of 4.5 g per mouse, the daily CLA intake was 0.045 g per mouse. Based on the Meeh‐Rubner formula (BSA(m2) = k × (body weight g)2/3 × 10–6, Kmouse = 9.1, Khuman = 10.1)。In contrast, the typical dosage of rumen‐protected CLA used in dairy cattle trials is usually 20–100 g per cow per day (equivalent to approximately 0.03–0.17 g/kg BW/day for a 600 kg cow) (Pappritz et al. 2011; Wei et al. 2024). The CLA exposure dose in mice is generally similar to that in cattle. However, differences exist, primarily due to fundamental differences in the digestive systems of these two animal species: as monogastric animals, mice allow CLA to directly enter the small intestine and be absorbed; whereas in ruminants like cows, rumen microbes strongly biohydrogenate unsaturated fatty acids, causing most unprotected CLA to be converted into stearic acid in the rumen, resulting in extremely low bioavailability (Jenkins and Bridges Jr. 2007; Bauman et al. 2020). Therefore, when extrapolating the findings of this study to dairy cattle, it is essential to use rumen‐protected CLA formulations, and dosages cannot simply be scaled according to body weight.

## 5 Conclusions

Our study showed that, compared with the CN group, dietary supplementation with 1% CLA enhances SOD activity and the T‐AOC while concurrently reducing the levels of MDA, GPx, and the pro‐inflammatory cytokines IL‐1β, TNF‐α, and IL‐6 in murine serum and mammary tissues. Compared with the CN group, TNF‐α expression levels in the 1% CLA group reached the lowest levels. The inflammatory infiltration in the 1% CLA group was reduced significantly. In conclusion, the diet containing 1% CLA showed the most significant antioxidant and anti‐inflammatory effects in the serum and mammary tissue of mice. This study employed mice as an animal model, thereby providing foundational data for the future application of CLA in dairy cow farming. In this study, mice were used as an animal model for dose optimization and efficacy verification in vivo, These results provide a theoretical basis for the application of CLA in dairy cow breeding to alleviate mammary gland inflammation.

## Author Contributions

**Zheming Ren:** writing – original draft, writing – review and editing, conceptualization, methodology, software, data curation, supervision, formal analysis, validation, investigation, visualization. **Wenqi Liang:** writing – review and editing, methodology, validation, visualization, software, formal analysis. **Hang Zhang:** writing – review and editing, project administration, funding acquisition, investigation, validation, visualization, formal analysis, methodology, data curation, supervision, resources, software, conceptualization. **Minghui Liu:** software, methodology, investigation. **Yuqiong Wang:** supervision, software, validation.

## Funding

This research was funded by the Natural Science Foundation of Inner Mongolia (No. 2023LHMS03060, No. 2023LHMS03009).

## Ethics Statement

The animal experiments were approved by the Animal Care and Ethics Committee of Inner Mongolia Minzu University (NO. NMD‐DW‐2024‐11‐15).

## Consent

The authors have nothing report.

## Conflicts of Interest

The authors declare no conflicts of interest.

## Data Availability

The data that support the findings of this study are available from the corresponding author upon reasonable request.
